# Supplementary material for: Iterative improvement in the automatic modular design of robot swarms
Source: PeerJ Comput Sci. 2020 Dec 7;6:e322. doi: 10.7717/peerj-cs.322 (PMC7924708; doi:10.7717/peerj-cs.322)
Supplement: Supplemental Information 3 [file peerj-cs-06-322-s003.zip › argos3/doc/api/standalone/a00635_source.html]

ARGoS: plugins/simulator/visualizations/qt-opengl/qtopengl\_application.cpp Source File


- Main Page
- Related Pages
- Namespaces
- Classes
- Files

- File List
- File Members

# plugins/simulator/visualizations/qt-opengl/qtopengl\_application.cpp

Go to the documentation of this file.

```
00001 
00007 #include "qtopengl_application.h"
00008 
00009 #include <argos3/core/utility/logging/argos_log.h>
00010 
00011 #include <typeinfo>
00012 
00013 namespace argos {
00014 
00015    /****************************************/
00016    /****************************************/
00017 
00018    bool CQTOpenGLApplication::notify(QObject* pc_receiver,
00019                                      QEvent* pc_event) {
00020       try {
00021          return QApplication::notify(pc_receiver, pc_event);
00022       } catch (std::exception& ex) {
00023          fprintf(stderr, "%s\n", ex.what());
00024          QApplication::exit(1);
00025       } catch (...) {
00026          qFatal("Error <unknown> sending event %s to object %s (%s)", 
00027                 typeid(*pc_event).name(),
00028                 qPrintable(pc_receiver->objectName()),
00029                 typeid(*pc_receiver).name());
00030       }
00031       /* Should never get here */
00032       return false;
00033    }
00034 
00035    /****************************************/
00036    /****************************************/
00037 
00038 }
```

---

Generated on 10 Jul 2018 for ARGoS by 
 1.6.1
